# Design and Performance Study of a Gradient Honeycomb Vibration-Damping Structure for the Knee Joint

**DOI:** 10.3390/biomimetics11010084

**Published:** 2026-01-22

**Authors:** Shucheng Lou, Li Feng

**Affiliations:** College of Home and Art Design, Northeast Forestry University, Harbin 150040, China; ztzaw0222@nefu.edu.cn

**Keywords:** vibration-damping structure, bio-inspired honeycomb, gradient design, knee joint gait

## Abstract

Excessive vibration during human knee joint movement poses challenges to biomechanical performance and comfort, which this study aims to mitigate through the design of a bio-inspired honeycomb-based vibration-damping structure, for the purpose of optimizing dynamic vibration absorption efficiency. Three honeycomb geometries—regular triangle, square, and regular hexagon—were evaluated via dynamic mechanical simulation, identifying the regular hexagon as the most effective base configuration. Using the control variable method within reasonable parameter ranges, finite element analysis was employed to systematically examine the influence of wall thickness, side length, and gradient of the regular hexagonal honeycomb on its damping performance. The findings demonstrate that vibration damping is maximized under a configuration with a wall thickness of 1.8 mm, a side length of 6 mm, and a gradient of 110%.

## 1. Introduction

The human knee joint undergoes dynamic impact loads several times the body weight during movement, which can easily lead to tissue damage and functional degeneration [1]. To effectively absorb and cushion impact energy during knee joint activity, vibration damping technology has become a key research focus. Current mainstream damping solutions mainly include spring structures, gas dampers, and honeycomb structures. Spring structures utilize elastic deformation to store and release energy, offering advantages such as simple structure and low cost. However, their stiffness adjustment capability is limited, making them prone to resonance under low-frequency impacts. Additionally, rigid contact with metal materials may cause secondary impact issues [2,3]. Gas dampers generate damping effects by compressing gas within a sealed chamber and are widely used in knee braces and prosthetic cushioning systems [4,5]. Nevertheless, these structures require high sealing performance, are generally bulky, and may exhibit significant damping performance degradation in low-temperature environments [6].

As a typical lightweight and high-strength porous material, honeycomb structures demonstrate significant application value and research potential in the field of vibration damping [7]. The performance of such structures is highly designable. Traditional design methods primarily involve adjusting parameters such as cell geometry, wall thickness, and arrangement to optimize macroscopic stiffness and energy absorption characteristics [8]. In recent years, with advances in design theory and manufacturing technology, topology optimization-based design methods for non-uniform material distribution have significantly expanded the functional possibilities of honeycomb structures. These methods can automatically generate optimal material layouts under specific constraints through algorithm-driven processes, resulting in new honeycomb configurations with high-performance indicators [9]. Against this backdrop, multi-scale simulation techniques, parametric modeling, and machine learning-assisted strategies are gradually being applied to the performance prediction and inverse design of honeycomb structures, further promoting the development of intelligent, high-performance damping structures [10,11]. Currently, honeycomb structures have been preliminarily explored and applied in areas such as human protection and equipment impact resistance, with their excellent energy management capabilities and structural adaptability holding broad prospects in biomechanical scenarios [12,13].

The loads borne by the knee joint during the gait cycle exhibit biomechanical characteristics such as dynamic time-variance, multi-directional composition, and significant individual differences, thereby imposing special requirements on the adaptability, tunability, and mechanical compatibility of damping structures [14]. Honeycomb structure design enables effective regulation of stiffness and energy absorption behavior, thereby matching the load variation patterns of the knee joint across different movement phases. It provides conformal cushioning under low-load conditions and stable support during high-impact phases, achieving graded load-bearing under dynamic responses [15,16]. Additionally, honeycomb structures possess excellent deformation coordination and elastic recovery capabilities. They can undergo coordinated deformation during joint flexion and extension without causing motion interference and maintain structural integrity and functional resetability after multiple loading cycles, avoiding adverse effects on the normal biomechanical function of the knee joint [17,18].

Despite significant progress in vibration damping research involving honeycomb structures, their application in knee joint protection remains in its early stages. Existing studies mostly focus on mechanical performance analysis under single-load conditions, lacking a systematic design addressing the complex motion environment of the knee joint. To address these issues, this paper proposes a novel honeycomb vibration-damping structure design method based on knee joint gait analysis. By integrating kinematic data and finite element simulations, the load characteristics of the knee joint during the gait cycle are quantified. Based on this, the geometric parameter distribution of the honeycomb is optimized to design a high-performance vibration-damping honeycomb structure, aiming to provide effective protection for the knee joint during movement.

## 2. Bio-Inspired Mechanical Analysis of Honeycomb Structures for Knee Joint Vibration Damping

During the initial contact (IC) phase of the gait cycle, the ground reaction force (GRF) generated at the instant of foot-ground contact is transmitted through the heel to the knee joint, creating a high-intensity impact load [19]. Without effective attenuation, such dynamic loading can lead to soft tissue damage and even degenerative diseases such as osteoarthritis over time [20]. Through evolution, organisms in nature have developed various exceptional energy management mechanisms. In particular, bone tissues, represented by the human tibia, achieve efficient dissipation of impact energy and redistribution of stress through their gradient macro- and micro-structures [21]. This biological gradient mechanism provides key bionic insights for the design of knee joint vibration-damping structures.

### 2.1. Bionic Basis of Stress Propagation and Wave Theory

From a biomechanical perspective, the human tibia exhibits distinct geometric and material gradients: its proximal cross-sectional area is larger than the distal end, and the elastic modulus transitions continuously from cortical to trabecular bone [22,23]. Such gradient structures allow stress waves entering from the distal end during the IC phase to be gradually dissipated through micro-buckling, plastic deformation, and multiple scattering within the trabeculae, significantly reducing the risk of damage due to stress concentration [24]. This mechanical behavior aligns with Saint-Venant’s principle, whereby localized high stresses gradually homogenize over propagation distance, demonstrating the superiority of biological systems in achieving intelligent energy redistribution through structural gradients.

Based on this mechanism, similar gradient designs can be introduced into artificial damping structures to replicate the energy dissipation pathways of biological systems in an in vitro bionic manner. To quantitatively describe stress attenuation along gradient structures, the following exponential decay model is established (after [21] with modification):

(1) $$\sigma (x)=\sigma_{0}\cdot e^{-\beta x}$$

where $\sigma_{0}$ is the initial contact stress and β is the attenuation coefficient, which depends on material density, elastic modulus, and gradient configuration. The model focuses on impact loading dominated by the vertical ground reaction force (vGRF) during gait, with the X-axis defined as the primary load-transfer direction along the tibial long axis. As the knee mainly sustains vertical impact during gait and the principal stress in the X-direction contributes most to tissue-injury risk [19,24], this study—serving as a foundational model for biomimetic design—prioritizes revealing stress-attenuation mechanisms along this primary loading axis. Subsequent analyses could be extended to multi-axial simulations. Moreover, the gradient structure of bone tissue has evolved primarily to optimize principal-stress transmission, with shear and other stresses being secondary—consistent with a core-function-driven biomimetic approach. Thus, a uniaxial stress model is adopted here for simplified analysis. This model indicates that stress decays exponentially with propagation distance, consistent with the “stress-shielding effect” observed in biological tissues [24]. To further account for the effect of material inhomogeneity on stress wave propagation, the one-dimensional wave equation may be used (after [3] with modification):

(2) $$\frac{\partial^{2}u}{\partial t^{2}}=c^{2}\frac{\partial^{2}u}{\partial x^{2}}$$

where u is displacement and $c=\sqrt{E/\rho }$ is the wave speed. In gradient structures, the elastic modulus E and density ρ vary with position, so the wave speed c should be treated as a position-dependent function c(x) to more accurately describe wave dispersion and reflection during propagation, thereby providing theoretical support for gradient structure design.

### 2.2. Energy Absorption and Dissipation Mechanisms in Gradient Structures

In terms of energy, the energy absorption capacity of a gradient structure can be represented by the area under the stress–strain curve (after [25] with modification):

(3) $$U_{d}=\int_{0}^{\varepsilon_{f}}\sigma (\varepsilon )d\varepsilon$$

For structures with gradient characteristics, the total energy absorption is the sum of the deformation energies across all layers (after [25] with modification):

(4) $$U_{total}=\sum_{i=1}^{n}U_{d,i}=\sum_{i=1}^{n}\int_{0}^{\varepsilon_{f,i}}\sigma_{i}(\varepsilon )d\varepsilon$$

This expression indicates that gradient structures can achieve sequential energy dissipation through the progressive collapse and deformation of cellular elements at different levels, thereby avoiding abrupt failure. This behavior is highly consistent with the mechanical transition mechanism of bone, demonstrating the significant advantage of bio-inspired gradient designs in enhancing structural reliability.

### 2.3. System Dynamic Modeling and Vibration Damping Performance Optimization

The knee joint equipped with a gradient honeycomb damping device can be simplified as a damped mass-spring system, with the equation of motion (after [2] with modification):

(5) $$m\overset{..}{x}+c\dot{x}+kx=F(t)$$

where m is the effective mass, c is the damping coefficient, k is the stiffness, and F(t) is the external excitation. By designing the geometric distribution, the gradient structure can modulate the equivalent stiffness $k_{eq}$ and damping coefficient $c_{eq}$ to optimize frequency response characteristics, thereby effectively suppressing resonance and attenuating vibration energy. The equivalent stiffness can be expressed as the second derivative of elastic potential energy with respect to deformation (after [8] with modification):

(6) $$k_{eq}=\frac{\partial^{2}U}{\partial \delta^{2}}$$

where U is the total deformation energy of the structure and δ is the generalized displacement. This expression provides an effective method for quantifying the stiffness of complex inhomogeneous structures and indicates that by adjusting the geometric distribution of the gradient structure, the stiffness and damping characteristics of the system can be optimized to effectively suppress vibration, reduce peak impact force, and improve damping performance.

Based on the above bionic principles and mechanical models, it can be concluded that the gradient mechanical behavior of biological bone tissue and honeycomb structures exhibits significant functional similarities in terms of energy dissipation paths and bending optimization. Through bio-inspired design, the resulting gradient honeycomb structure can mimic the continuous stiffness variation of bone, achieving graded pressure bearing and efficient energy absorption under dynamic loading. Therefore, this study aims to design a novel wearable gradient honeycomb damping device for the knee joint. This device simulates and enhances the natural stress attenuation and energy dissipation mechanisms externally, with the goal of effectively reducing the peak impact force transmitted to the knee joint.

## 3. Data Acquisition from Plantar Pressure Experiments in Humans

### 3.1. Experimental Procedure

The subject recruitment followed the Chinese National Standard Human Dimensions of Chinese Adults (GB/T 10000-2023) [26]. One healthy adult male (age: 24 years, height: 178 cm, weight: 70 kg) was selected, with no history of neurological, muscular, or skeletal disorders, and no lower limb injuries within the past six months. The subject was instructed to avoid strenuous exercise for 24 h prior to the experiment. The study was approved by the Ethics Committee of Northeast Forestry University, and written informed consent was obtained from the participant. The procedures and potential risks were explained in detail, and the subject was free to withdraw at any time.

In this experiment, plantar pressure data during natural walking were collected using a high-resolution body pressure measurement system (BIMS, Tekscan Inc., Norwood, MA, USA). The system employs a piezoresistive sensor array, in which each sensor consists of a polyester film coated with a special semiconductive resistive ink. The electrical resistance of the sensor varies proportionally with the applied pressure, enabling accurate conversion of pressure into an electrical signal. Data acquisition was performed in real time using the accompanying CONFORMat Research 7.60 software. With high spatial resolution and a sampling frequency of 100 Hz, the system ensures timely and accurate capture of dynamic pressure data, providing a reliable foundation for gait analysis.

The experiment was conducted in an indoor corridor (15 m long, 2.5 m wide) at the College of Furnishings and Art Design, Northeast Forestry University. The floor was flat and smooth, and environmental conditions were controlled at 23 °C and 50% relative humidity to minimize external interference.

Before the formal experiment, the subject changed into standardized experimental attire. The experimental procedure and precautions were explained in detail, and a 5 min adaptation period was provided to familiarize the subject with the environment and walking requirements. The subject stood in the preparation area, looked straight ahead, and walked at a natural pace through the force measurement area until reaching the stop zone. Data recording was terminated only after the subject completely stepped off the force plate. To ensure data reliability and repeatability, the subject performed 20 valid walking trials with a 1 min rest between trials to prevent muscle fatigue from affecting gait patterns.

### 3.2. Experimental Results

A high-resolution body pressure measurement system (Tekscan, Norwood, MA, USA) was used to successfully collect plantar pressure dynamics during natural walking. A total of 20 raw time–pressure sequences were initially acquired. Strict quality control was applied: invalid data exhibiting sensor drift, transient signal loss, or obvious gait adjustments were excluded based on visual screening and biomechanical rationality criteria. Furthermore, based on the characteristic biphasic pattern of vertical ground reaction force (vGRF) in healthy adult gait [14], two trials with abnormal vGRF curves were discarded. Ultimately, 18 valid trials were retained for subsequent analysis, yielding an effective data rate of 90%.

**Figure 1:**
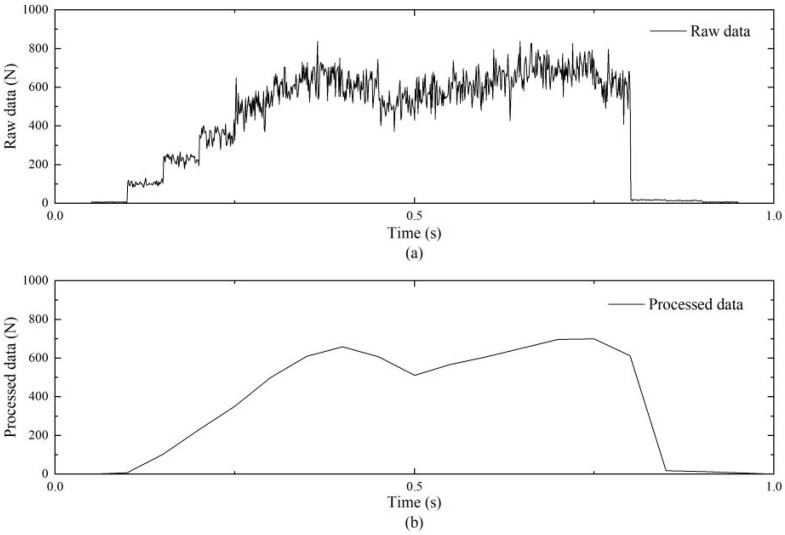
(**a**) Raw plantar pressure data; (**b**) Processed plantar pressure data.

To enhance data consistency and comparability, a 1 s interval containing a complete gait cycle was extracted from each trial. Raw readings were exported using CONFORMat Research 7.60 software. For subsequent analysis, the raw data from all valid trials were segmented and averaged at 0.05 s intervals, significantly reducing data variability while preserving key dynamic characteristics. Figure 1 shows a comparison between the raw and the processed plantar pressure data.

**Figure 2:**
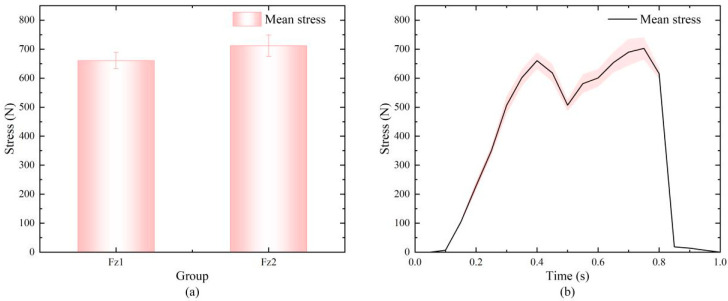
(**a**) Analysis of the Fz1 and Fz2 data; (**b**) Averaged plantar pressure across valid trials. The red area indicates the distribution of plantar pressure values at this time.

The precise values of the first (Fz1) and second (Fz2) peak forces were obtained by extracting peaks from 18 sets of valid experimental data. First, the normality of both peak force datasets was assessed using the Shapiro–Wilk test, which is suitable for the present small sample size (n = 18). The results showed that Fz1 (W = 0.907, *p* = 0.077 > 0.05) and Fz2 (W = 0.951, *p* = 0.447 > 0.05) both followed a normal distribution, satisfying the prerequisite for paired-sample *t*-tests. For standardized comparison, the raw pressure data (in N) were normalized to body weight (BW) multiples based on the subject’s weight (70 kg, i.e., 686 N) for subsequent analysis. The calculated results showed that the mean value of Fz1 was 1.02 ± 0.05 BW, ranging from 0.90 to 1.05 BW, while that of Fz2 was 1.05 ± 0.06 BW, ranging from 0.94 to 1.13 BW. To rigorously examine the difference between the Fz1 and Fz2 peak forces, a paired-sample *t*-test was performed. Based on the exact peak force values, the results (also shown in Figure 2a) indicated that Fz2 was significantly greater than Fz1 (t(17) = −5.157, *p* < 0.001). The mean paired difference was −51.40 N. Furthermore, Cohen’s d effect size was calculated as 1.215, indicating a large practical significance of the difference between Fz1 and Fz2.

Figure 2b shows the averaged time–pressure curve from the 18 valid trials. The vGRF curve clearly exhibits the classic “biphasic” pattern characteristic of healthy adult gait. Precise analysis of the averaged curve revealed that the first peak (Fz1) occurred during the early stance phase (approximately 28% of the gait cycle), corresponding to the heel-strike impact. The second peak (Fz2) occurred during the late stance push-off phase (approximately 68% of the gait cycle), primarily resulting from the lever effect of ankle plantar flexion and the propulsive force generated by concentric contraction of the triceps surae [27]. A distinct valley between the two peaks corresponds to the full-foot support phase, where vGRF briefly decreases, indicating effective buffering of impact energy by the musculoskeletal system.

## 4. Simulation Experiments and Results

Based on the plantar pressure experimental results obtained in Section 3.2, the averaged vGRF time-history curve (Figure 2b) was converted into the load boundary condition of the finite element model to accurately reflect the actual loading state of the knee joint in the simulation. Specifically, the load was defined as an axial dynamic concentrated force applied to the distal end of the tibia in a simplified lower-limb model. The force-time curve matched the experimentally measured vGRF curve (expressed in body weight multiples, BW), thereby simulating the impact load transmitted from the foot to the knee joint during the gait cycle.

### 4.1. Design of the Honeycomb Vibration-Damping Structure

Honeycomb structures are valued in biomedical protection for their lightweight nature, high specific strength, and excellent dynamic energy absorption properties. Their core advantage lies in the ability to synergistically regulate mechanical response and energy management efficiency through cell optimization. Existing studies confirm that regular polygonal honeycombs (triangular, quadrilateral, and hexagonal) outperform other complex configurations in “reducing peak stress and maintaining a stable plateau” [25]. Therefore, this study focuses on these three typical configurations for subsequent experiments.

**Figure 3:**
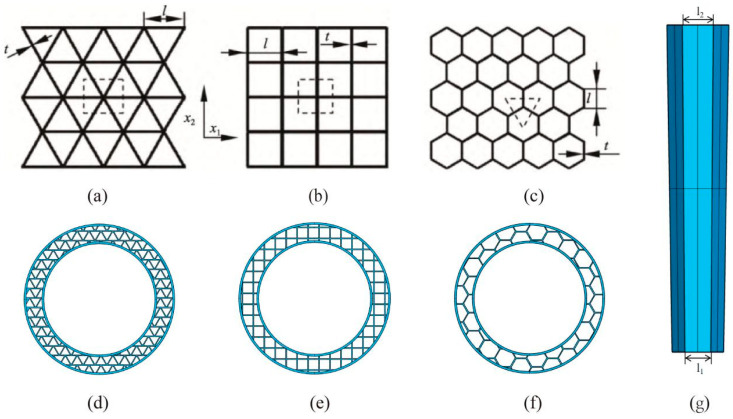
(**a**) Regular triangular honeycomb; (**b**) Regular quadrilateral honeycomb; (**c**) Regular hexagonal honeycomb; (**d**) Triangular damping structure; (**e**) Quadrilateral damping structure; (**f**) Hexagonal damping structure; (**g**) Front view of the honeycomb cell within the vibration-damping structure.

Drawing on existing literature regarding finite element modeling of two-dimensional porous materials, this study established finite element models for regular triangular, square, and hexagonal honeycomb structures. As shown in Figure 3, in the models, the cell edge length was set to 1, and the wall thickness was denoted as “t”. To further optimize structural performance and accommodate practical application scenarios, a gradient design was introduced: the side length of the bottom-layer honeycomb cells adjacent to the ground, denoted as l_1_, was kept constant, while the ratio of the top-layer cell side length l_2_ to l_1_ was defined as the gradient n, thereby enabling graded variation in structural performance. (In the finite element model of this study, for unified definition and simplified modeling, the honeycomb cell side length l refers to the distance between the centerlines of adjacent parallel cell walls, i.e., the dimension based on the mid-thickness of the walls.)

To better fit the anatomical shape of the human knee joint and improve wearing comfort, the honeycomb damping structure was designed in a ring shape. Furthermore, to avoid discomfort caused by stress concentration on the skin and soft tissues, buffer layers were applied to the inner and outer edges as well as the upper and lower surfaces of the honeycomb structure. This effectively disperses local stress and enhances the overall cushioning performance of the structure, providing more comprehensive protection for the knee joint during movement. Based on anthropometric data (the average calf circumference of Chinese males is 350.4 mm [28]), the inner diameter of the annular structure was set to 56 mm. Key geometric parameters for configuration comparison are listed in Table 1.

### 4.2. Experiment for Determining the Honeycomb Damping Structure Shape

#### 4.2.1. Finite Element Modeling Details

To simplify the computational process while ensuring evaluation accuracy, a simplified lower-limb model was employed for analysis using the commercial finite element software Abaqus/Standard (Dassault Systèmes, Vélizy-Villacoublay, France). This model abstracts the morphology of muscles and bones into cylindrical solid models based on anthropometric percentile data, preserving their key mechanical characteristics.

A mesh sensitivity study was performed to balance computational accuracy and cost. The honeycomb damping structure was meshed using 10-node modified quadratic tetrahedral elements (C3D10M), suitable for complex geometries and contact analysis. The lower-limb structure was discretized with 8-node linear reduced-integration hexahedral elements (C3D8R). The total number of nodes and elements for the lower-limb skeletal-muscle model was 22,787 and 18,997, respectively. The node and element counts for the regular hexagonal, square, and equilateral triangular honeycomb structures were 59,557/40,901, 73,062/51,046, and 104,329/59,078, respectively.

Silicone was selected for the honeycomb structure due to its excellent energy dissipation properties, biocompatibility, and conformability. It was modeled as an isotropic linear elastic material. The lower-limb bones were also defined as isotropic linear elastic materials, while the muscle soft tissue was modeled to represent its compliant behavior. All material parameters used are listed in Table 2.

A general contact algorithm was used to define interactions between bone-muscle, bone-plantar tissue, and muscle-honeycomb structure. The tangential behavior was set with a penalty friction coefficient of 0.2, and the normal behavior was defined as “hard” contact, allowing separation after contact. A tie constraint was applied between the muscle and plantar soft tissue. The loading consisted of two parts: (1) A uniform pressure of 2 kPa was applied perpendicularly to the inner surface of the damping structure ring to simulate the comfortable pressure from lower-limb garments. (2) The processed plantar pressure data (the averaged vGRF curve from Section 3.2) was applied as a vertical upward ground reaction force on the bottom surface of the foot model to simulate the loading during the gait cycle. The top and bottom surfaces of the simplified femur model were set as fixed boundary conditions (all degrees of freedom constrained).

#### 4.2.2. Determination of the Optimal Honeycomb Configuration

To accurately evaluate the vibration-damping performance of the honeycomb structures, the experiment was divided into a control group and experimental groups. The control group simulated the normal human gait cycle without the honeycomb damping structure. Experimental groups 1, 2, and 3 simulated the gait cycle while wearing the equilateral triangular, square, and regular hexagonal honeycomb damping structures, respectively.

**Figure 4:**
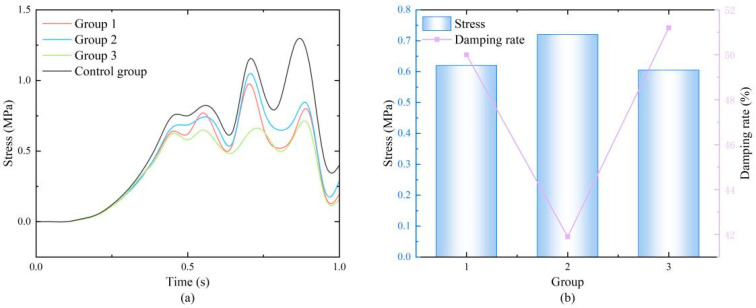
(**a**) Stress variation diagrams of honeycomb structures with different configurations; (**b**) Numerical values at the moment of maximum stress and the damping ratios.

Finite element experiments were conducted to obtain the stress variation in the human lower limb bones, with the average stress on the upper end of the femur as the core indicator. The results are shown in the figures. The femoral stress curve in Figure 4a exhibits more fluctuating peaks than the plantar pressure curve in Figure 2b. This is primarily because the plantar pressure curve represents the external load input, whereas the femoral stress is the dynamic response after the load is transmitted through the coupled system of lower-limb bone–soft tissue–honeycomb damping structure. As an elastodynamic system, the finite element model generates inherent vibrational responses under time-varying load excitation, thereby introducing additional fluctuations into the stress time history. This is a common phenomenon in dynamic simulations and reflects the vibrational characteristics of the structure under realistic dynamic conditions. Figure 4a shows the stress variation curves of the four groups over a complete gait cycle. Overall, the stress trends of all experimental groups were consistent with the control group, all showing a global maximum stress peak around 0.85 s and secondary peaks around 0.55 s and 0.75 s, which aligns with the typical load characteristics of the knee joint during walking. The stress amplitudes of the experimental groups were significantly reduced, indicating that all three honeycomb structures provided good cushioning effects.

To further quantify and compare the damping efficiency of different configurations, detailed data at the time of maximum stress (0.85 s) were extracted and plotted in Figure 4b. The stress value of the control group at this moment was as high as 1.237 MPa. In contrast, the stress values for the regular triangular, square, and hexagonal honeycomb structures at this moment were reduced to 0.620 MPa, 0.720 MPa, and 0.605 MPa, respectively. Using the damping ratio formula:

(7) $$\eta =\left(1-\frac{\sigma_{experimental\;group}}{\sigma_{control\;group}}\right)\times 100\%$$

The calculated damping efficiencies were 49.9%, 41.8%, and 51.1%, respectively. The hexagonal configuration exhibited the best peak stress reduction capability. Furthermore, analysis of the full time-history data indicated that the hexagonal honeycomb maintained the lowest stress levels at most time points, and its stress curve fluctuations were the smoothest. This suggests that this configuration not only effectively handles instantaneous impact loads but also provides more stable and continuous protection throughout the movement cycle, potentially significantly reducing the risk of cumulative fatigue damage. Therefore, the regular hexagon was selected as the core configuration for the subsequent honeycomb damping system, and further structural parameter optimization studies were based on this selection.

### 4.3. Single-Factor Simulation Experiments on the Bio-Inspired Honeycomb Structure

Based on the determined regular hexagonal honeycomb configuration, this study further conducted single-factor simulation experiments to systematically investigate the influence of three key geometric parameters—wall thickness (“t”), edge length (“l”), and gradient (“n”)—on its damping performance, aiming to provide precise parameter value ranges for subsequent multi-parameter collaborative optimization.

#### 4.3.1. Influence of Wall Thickness on the Damping Performance of the Regular Hexagonal Honeycomb Structure

To investigate the effect of cell wall thickness (t) on energy absorption characteristics, this study fixed the side length l = 6 mm and the gradient n = 100%, and selected five wall thicknesses—1.0, 1.2, 1.4, 1.6, and 1.8 mm—for finite element simulation. The selection of wall thickness parameters was based on practical considerations for knee-joint vibration-damping applications: an excessively small wall thickness may lead to structural instability or manufacturing challenges, whereas an overly large thickness would significantly increase structural weight, reduce flexibility, and compromise wearing comfort. Therefore, thicknesses ranging from 1.0 to 1.8 mm were chosen at intervals of 0.2 mm to systematically evaluate the trend of wall-thickness influence on performance. This parameter range is comparable to those used in related studies on the fabrication and mechanical behavior of thin-walled components [30].

**Figure 5:**
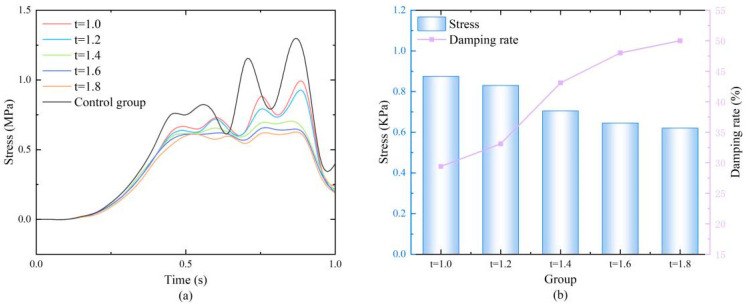
(**a**) Stress variation diagrams of honeycomb structures with different wall thicknesses; (**b**) Numerical values at the moment of maximum stress and the damping ratios.

The results (Figure 5a) showed that the stress time-history curves of all experimental groups followed the same trend as the control group, but the stress amplitudes were significantly reduced. In-depth analysis at the peak stress moment (0.85 s) (Figure 5b) revealed that the stress in the control group was as high as 1.237 MPa. As the wall thickness increased, the peak stress showed a clear monotonic decreasing trend: with wall thicknesses of 1.0, 1.2, 1.4, 1.6, and 1.8 mm, the corresponding peak stresses were 0.875, 0.83, 0.705, 0.645, and 0.62 MPa, and the damping ratios were 29.3%, 32.9%, 43.0%, 47.8%, and 49.9%, respectively. This phenomenon stems from the fact that increased wall thickness significantly enhances the bending stiffness of the cell walls and the compressive strength of the overall structure, thereby improving the energy absorption capacity. This allows more impact energy to be dissipated through elastic deformation and plastic yielding of the material rather than being transmitted to the bone. It is worth noting that when the wall thickness increased from 1.6 mm to 1.8 mm, the improvement in damping ratio (2.1%) was much smaller than the increase from 1.4 mm to 1.6 mm (4.8%), indicating diminishing returns on performance enhancement with increasing wall thickness, necessitating a trade-off between material usage and comfort. Therefore, considering both damping effectiveness and engineering applicability, the wall thickness parameter range for subsequent orthogonal experiments was determined to be 1.4–1.8 mm.

#### 4.3.2. Influence of Edge Length on the Damping Performance of the Regular Hexagonal Honeycomb Structure

To investigate the effect of cell edge length (“l”) on damping performance, this study fixed the wall thickness “t” = 1.4 mm and gradient “n” = 100%, and selected five edge lengths: 2, 4, 6, 8, and 10 mm for simulation.

**Figure 6:**
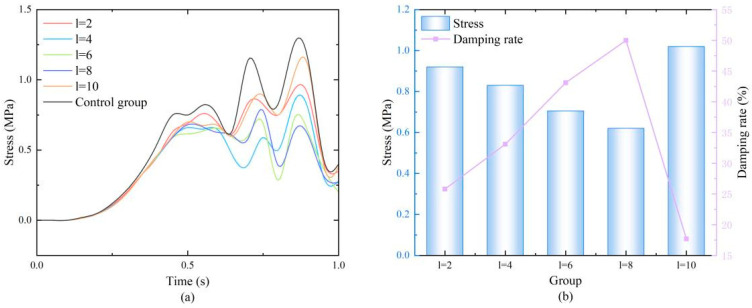
(**a**) Stress variation diagrams of honeycomb structures with different edge lengths; (**b**) Numerical values at the moment of maximum stress and the damping ratios.

The results (Figure 6a) indicated that the influence of edge length on performance was not monotonic but had an optimal range. The relationship between peak stress (at 0.85 s) and edge length was as follows: for edge lengths of 2, 4, 6, 8, and 10 mm, the peak stresses were 0.92, 0.83, 0.705, 0.62, and 1.02 MPa, and the corresponding damping ratios were 25.6%, 32.9%, 43.0%, 49.9%, and 17.5%, respectively. Data analysis revealed a key trend: damping performance first improved and then deteriorated with increasing edge length. When the edge length was too small (e.g., 2 mm), the honeycomb structure was too weak to effectively resist impact loads. When the edge length increased to 4–8 mm, the structural stiffness and energy absorption capacity were well matched, resulting in optimal performance (the damping ratio peaked at 8 mm, reaching 49.9%). However, when the edge length was too large (e.g., 10 mm), the excessively large unit cell size made the local structure too rigid, compromising the excellent compressibility of the honeycomb material, exacerbating stress concentration, and causing a sharp decline in cushioning performance. Therefore, the edge length parameter range for subsequent orthogonal experiments was determined to be 4–8 mm to cover the peak performance interval.

#### 4.3.3. Influence of Gradient on the Damping Performance of the Regular Hexagonal Honeycomb Structure

To investigate the effect of gradient (“n”, defined as the ratio of top to bottom cell edge length) on performance, this study fixed the wall thickness “t” = 1.4 mm and edge length “l” = 6 mm, and selected five gradients: 80%, 90%, 100%, 110%, and 120% for simulation.

**Figure 7:**
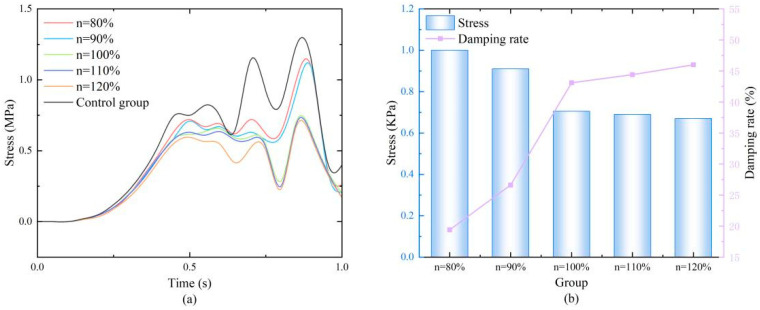
(**a**) Stress variation diagrams of honeycomb structures with different gradients; (**b**) Numerical values at the moment of maximum stress and the damping ratios.

Gradient design aims to optimize the stress transmission path through the spatial distribution of structural stiffness. The results (Figure 7) showed that gradient variation significantly affected damping performance. At the peak stress moment (0.85 s), the stress values corresponding to gradients of 80%, 90%, 100%, 110%, and 120% were 1.00, 0.91, 0.705, 0.69, and 0.67 MPa, respectively, and the damping ratios were 19.2%, 26.4%, 43.0%, 44.2%, and 45.8%, respectively. The results demonstrated that increasing the gradient within a certain range (“n” > 100%), i.e., reducing the top cell size, effectively improved damping performance. This is because a “top-down” increasing stiffness gradient better conforms to the diffusion path of impact loads, facilitating smoother attenuation of stress waves within the structure and avoiding stress concentration caused by sudden stiffness changes. When the gradient increased from 100% to 120%, the performance gain tended to plateau. Considering manufacturability, the gradient parameter range for subsequent orthogonal experiments was determined to be 100–120%.

### 4.4. Orthogonal Simulation Experiments on the Bio-Inspired Honeycomb Structure

This study employed an orthogonal experimental design to perform a multi-factor collaborative optimization analysis of the three key parameters: wall thickness (“t”), edge length (“l”), and gradient (“n”). Based on the parameter ranges determined from the single-factor experiments, specific parameter values were selected using an evenly distributed approach. As shown, the dynamic responses of 9 experimental groups during the lower limb gait cycle were obtained through finite element simulation. The damping ratio (η) at the peak stress moment (0.85 s) was used as the evaluation indicator, with a higher η indicating better comprehensive damping performance. The orthogonal experimental scheme and results are shown in Table 3.

A range analysis was performed on the vibration-reduction results obtained from the orthogonal experiments, and the results are presented in Table 4. Here, K_i_ denotes the sum of the vibration-reduction ratios for all experimental results corresponding to the *i*-th level of a given factor; k_i_ represents the average vibration-reduction ratio at that level (k_i_ = K_i_/s, where the number of replicates per level in this case is s = 3); and T is the range of the k_i_ values for that factor, reflecting the degree of influence of the factor on the damping ratio.

The magnitude of the range (T) directly reflects the degree of influence of each factor on the damping ratio. Based on the precise calculations in Table 2, the order of factors affecting damping performance is: wall thickness “t” (T = 13.07) > gradient “n” (T = 7.82) > edge length “l” (T = 7.55). This result indicates that wall thickness (“t”) is the most significant factor influencing damping performance, with its range being substantially larger than the other two factors. This is because wall thickness directly determines the bending stiffness of the cell walls and the compressive strength of the overall structure, playing a dominant role in the energy absorption capacity of the honeycomb structure. The influence of gradient (“n”) and edge length (“l”) is similar, ranking as the second and third factors, respectively; they primarily affect performance by adjusting the stiffness distribution and cell density of the structure.

The optimal level for each factor should be the level corresponding to the maximum value of k_i_. Analysis of Table 2 yields:

Wall thickness “t”: k_3_ (50.28%) > k_2_ (50.15%) > k_1_ (37.22%), optimal level is level 3 (1.8 mm);

Edge length “l”: k_2_ (49.34%) > k_1_ (46.51%) > k_3_ (41.79%), optimal level is level 2 (6 mm);

Gradient “n”: k_2_ (49.34%) > k_3_ (46.78%) > k_1_ (41.52%), optimal level is level 2 (110%).

Therefore, the theoretically optimal parameter combination is “t_3_””l_2_””n_2_”, i.e., wall thickness 1.8 mm, edge length 6 mm, gradient 110%.

## 5. Discussion

The results of this study demonstrate that the designed biomimetic gradient honeycomb structure, particularly with a regular hexagonal topology and optimized geometric parameters (wall thickness: 1.8 mm, side length: 6 mm, gradient: 110%), can significantly attenuate the impact forces transmitted to the knee joint during gait. This finding validates the initial hypothesis that a structure mimicking the stress-diffusion gradient principle observed in natural bone tissue (such as the tibia) can effectively enhance energy dissipation capacity. The superior performance exhibited by the hexagonal configuration is consistent with and extends existing conclusions on the mechanical behavior of cellular solids. This configuration achieves an optimal balance among symmetry, in-plane stiffness, and stable plateau stress under compressive loading. Compared to triangular or square configurations, such a balance facilitates more uniform stress distribution and an orderly sequence of cell collapse within the structure, thereby improving overall energy absorption efficiency. Furthermore, the femoral stress curve obtained from the simulation (Figure 4a) exhibited more oscillatory peaks than the experimentally measured plantar pressure curve (Figure 1 and Figure 2). This discrepancy stems from the transfer characteristics of the dynamic system: plantar pressure represents an external excitation, while bone stress is an internal response filtered and amplified by the lower-limb biomechanical system (including bone, soft tissue, and the attached damping structure). Under transient loading, the finite element model excites dynamic structural responses, leading to richer frequency components in the stress time history. This aligns with the vibrational behavior of elastic bodies under impact loading and confirms the capability of the simulation to capture the dynamic response of the system. The achieved reduction in transmitted force validates the design approach framed by the dynamic system model (Equation (5)). While the optimal geometric parameters (t, l, n) were not translated into explicit time-varying coefficients k(t) and c(t), their combined effect successfully tuned the system’s dynamic impedance, minimizing the response (bone stress) to the gait-induced excitation F(t).

The parameter optimization results offer deeper mechanical insights. The dominant influence of wall thickness on damping ratio underscores its primary role in defining the fundamental bending stiffness and strength of the cellular walls. The positive effect of a positive gradient (n > 100%), where cell size decreases from the load-input surface, corroborates the bionic principle derived from bone’s macro-architecture. This gradient effectively creates a “soft” interface for initial contact, mitigating stress concentration, and a progressively stiffer interior to support higher loads, thereby optimizing the stress wave propagation path. This bridges our specific design with broader concepts in functionally graded materials for impact protection.

The implications of this work extend beyond knee joint protection. The developed design framework and findings are relevant for engineering applications requiring lightweight, tunable damping solutions, such as in sports equipment, aerospace seating, or precision instrument isolation. The successful integration of biomechanical gait data with parametric finite element analysis and orthogonal experimental design also presents a replicable methodology for the performance-driven design of other bio-inspired protective structures.

However, this study also has certain limitations, which highlight directions for future research. The current finite element simulations were based on assumptions of material isotropy and quasi-static loading, while the actual biomechanical environment involves material viscoelasticity, rate dependency, and complex multi-axial dynamic loading. Future work should therefore include dynamic mechanical testing of 3D-printed structural prototypes under physiologically relevant loading rates to validate the accuracy of the numerical predictions. Furthermore, exploring a wider design space via machine learning-assisted topology optimization could uncover non-intuitive, high-performance architectures. Long-term durability, fatigue resistance, and subject-specific customization based on varied anthropometric and gait data are crucial next steps for clinical or practical translation.

## 6. Conclusions

In response to the demand for knee joint vibration damping, this study successfully developed and optimized a knee joint vibration-damping structure based on a regular hexagonal gradient honeycomb through a systematic research approach combining bionic design, experimental validation, and numerical simulation. The main conclusions are as follows:

1. The dynamic loading characteristics during the gait cycle of a healthy adult were accurately obtained based on plantar pressure experiments. The vertical ground reaction force (vGRF) exhibited a typical bimodal pattern, with peak forces of 1.02 BW and 1.05 BW, providing accurate biomechanical boundary conditions for subsequent finite element simulations.
2. A comparative analysis of different topologies revealed that the regular hexagonal honeycomb demonstrated the optimal overall vibration-damping performance among the three candidate designs. It achieved a damping ratio of 51.1% at the peak stress moment and exhibited the most stable stress response throughout the entire gait cycle, confirming the advantages of its symmetrical structure and efficient energy absorption mechanism.
3. Single-factor and orthogonal experiments elucidated the influence of three key parameters—wall thickness, edge length, and gradient—on the damping performance. The order of their influence was identified as wall thickness > gradient > edge length. The theoretically optimal parameter combination was determined to be a wall thickness of 1.8 mm, an edge length of 6 mm, and a gradient of 110%. This combination achieves excellent damping performance (damping ratio > 53%) while also offering good engineering feasibility and potential for wearing comfort.

This study provides a theoretical basis and data support for the design of knee joint vibration-damping structures and validates the application potential of gradient bio-inspired honeycomb structures in the field of biomechanical energy absorption.

## Figures and Tables

**Table 1:** Design dimensions of the regular polygonal honeycomb vibration-damping structure.

Item	Dimension (mm)
Cell wall length	10
Cell wall thickness	1
Height of the honeycomb structure	38
Thickness of the damping structure	20
Thickness/height of the buffer layer	2

**Table 2:** Material properties of the lower-limb bones, muscle soft tissue, and plantar soft tissue [29].

Material	Density (t/mm^3^)	Young’s Modulus (MPa)	Poisson’s Ratio
Silicone	1.2 × 10^−9^	2.14	0.48
Lower-limb bones	1.9 × 10^−9^	7300	0.3
Lower-limb muscle soft tissue	1.1 × 10^−9^	0.15	0.45
Plantar soft tissue	1.06 × 10^−9^	0.45	0.49

**Table 3:** Damping performance results obtained from the orthogonal experiments.

Exp. No.	Wall Thickness	Edge Length	Gradient	Damping Ratio
	t (mm)	l (mm)	n (%)	η (%)
1	1.4 (1)	4 (1)	100 (1)	32.9
2	1.4 (1)	6 (2)	110 (2)	44.2
3	1.4 (1)	8 (3)	120 (3)	34.5
4	1.6 (2)	4 (1)	110 (2)	54.7
5	1.6 (2)	6 (2)	120 (3)	53.9
6	1.6 (2)	8 (3)	100 (1)	41.8
7	1.8 (3)	4 (1)	120 (3)	51.9
8	1.8 (3)	6 (2)	100 (1)	49.9
9	1.8 (3)	8 (3)	110 (2)	49.1

**Table 4:** Results of the range analysis.

	Wall Thickness	Edge Length	Gradient
	t (mm)	l (mm)	n (%)
K1	114.6	139.5	124.6
K2	150.4	148.0	148.0
K3	150.9	125.4	140.3
k1	37.2	46.5	41.5
k2	50.1	49.3	49.3
k3	50.3	41.8	46.8
T	13.1	7.5	7.8

## Data Availability

The original contributions presented in this study are included in the article material. Further inquiries can be directed to the corresponding author.
